# Directional soliton and breather beams

**DOI:** 10.1073/pnas.1821970116

**Published:** 2019-04-26

**Authors:** Amin Chabchoub, Kento Mozumi, Norbert Hoffmann, Alexander V. Babanin, Alessandro Toffoli, James N. Steer, Ton S. van den Bremer, Nail Akhmediev, Miguel Onorato, Takuji Waseda

**Affiliations:** ^a^Centre for Wind, Waves and Water, School of Civil Engineering, The University of Sydney, Sydney, NSW 2006, Australia;; ^b^Department of Ocean Technology Policy and Environment, Graduate School of Frontier Sciences, The University of Tokyo, Kashiwa, Chiba 277-8563, Japan;; ^c^Dynamics Group, Hamburg University of Technology, 21073 Hamburg, Germany;; ^d^Department of Mechanical Engineering, Imperial College London, London SW7 2AZ, United Kingdom;; ^e^Department of Infrastructure Engineering, The University of Melbourne, Parkville, VIC 3010, Australia;; ^f^School of Engineering, University of Edinburgh, Edinburgh EH9 3FB, United Kingdom;; ^g^Department of Engineering Science, University of Oxford, Oxford OX1 3PJ, United Kingdom;; ^h^Research School of Physics and Engineering, The Australian National University, Canberra, ACT 2600, Australia;; ^i^Dipartimento di Fisica, Università degli Studi di Torino, 10125 Torino, Italy;; ^j^Istituto Nazionale di Fisica Nucleare, Sezione di Torino, 10125 Torino, Italy

**Keywords:** nonlinear waves, solitons, directional localizations, extreme events

## Abstract

Solitons and breathers are nonlinear modes that exist in a wide range of physical systems. They are fundamental solutions of a number of nonlinear wave evolution equations, including the unidirectional nonlinear Schrödinger equation (NLSE). We report the observation of slanted solitons and breathers propagating at an angle with respect to the direction of propagation of the wave field. As the coherence is diagonal, the scale in the crest direction becomes finite; consequently, beam dynamics form. Spatiotemporal measurements of the water surface elevation are obtained by stereo-reconstructing the positions of the floating markers placed on a regular lattice and recorded with two synchronized high-speed cameras. Experimental results, based on the predictions obtained from the (2D + 1) hyperbolic NLSE equation, are in excellent agreement with the theory. Our study proves the existence of such unique and coherent wave packets and has serious implications for practical applications in optical sciences and physical oceanography. Moreover, unstable wave fields in this geometry may explain the formation of directional large-amplitude rogue waves with a finite crest length within a wide range of nonlinear dispersive media, such as Bose–Einstein condensates, solids, plasma, hydrodynamics, and optics.

## Significance

Understanding the fundamental dynamics of directional and localized waves is of significant importance for modeling ocean waves as well as predicting extreme events. We report a theoretical framework, based on the universal (2D + 1) nonlinear Schrödinger equation, that allows the construction of slanted solitons and breathers on the water surface. Our corresponding wave flume observations emphasize and uniquely reveal that short-crested localizations can be described as a result of nonlinear wave dynamics, complementing the linear superposition and interference arguments as has been generally suggested for directional ocean waves.

Ocean waves are complex 2D dynamical structures that cannot be easily modeled in their full complexity. Variations of depth, wind strength, and wave breaking; randomness; and large-amplitude waves add tremendously to this complexity (1). Despite these complications, the research on water waves is important and significant advances have been made so far (2). The progress is mainly due to simplified models that are used to analyze their dynamics (3). Moreover, validity of these models can be confirmed in down-scaled experiments in water wave facilities that exist in many research laboratories around the world. These experiments are crucially significant to build our understanding of larger-scaled oceanic waves. Evolution equations and their solutions are essential for water wave modeling, while computerized equipment is key for their accurate generation.

One of the essential complications in ocean wave dynamics is the unavoidable existence of two horizontal spatial coordinates. Directional behaviors of the surface waves in nature are of principal importance for practical applications ranging from wave forecast through modeling air–sea interactions to, most importantly, environmental and optical sciences. In a simplified way, such a wave field consists of many waves crossing each other at various angles, implying at a linear level that the water surface is a mere interference of short- and long-crested waves coming from different directions (4–6). Here, we leave aside these complexities. Instead, we start with a simple question: What does the second coordinate add to the dynamics when the waves are mostly unidirectional? This simple question must be answered before considering more complicated cases.

Indeed, unidirectional nonlinear wave dynamics on the water surface in deep water, that is, assuming that the water depth is significantly larger than the waves’ wavelength, can be described by the nonlinear Schrödinger equation (NLSE) that takes into account dispersion and nonlinearity (7). Being an integrable evolution equation, it allows for the study of particular and localized coherent wave patterns, such as solitons and breathers (8–10). The latter are of major relevance to study the fundamental wave dynamics in nonlinear dispersive media with a wide range of applications (11–13). While the NLSE has been formulated for planar waves and wave packets propagating in the same direction as the underlying carrier waves, there is also a generalization of the framework, the so-called directional NLSE, which allows the envelope and homogeneous planar carrier wave to propagate at an angle to each other. This possibility adds unexpected features to well-known nonlinear and coherent wave propagation motions as we examine in this work. Unfortunately, from the theoretical perspective, the directional deep-water NLSE is not integrable. As a consequence, these nontrivial nonlinear solutions are not easy to identify. Early attempts to generate some nonlinear states were based on symmetry considerations (14). It has been shown (15, 16) that each unidirectional solution of the NLSE has a family counterpart solution for which the packet beam propagates obliquely to the short-crested carrier wave.

These types of wave processes are directly relevant in oceanography (17–20). However, taking into account many areas in physics for which the NLSE is the fundamental governing equation, our ideas can be bluntly expanded to fields such as solids, Bose–Einstein condensates, plasma, and optics (21–25).

In the present study, we report an experimental framework and observations of hydrodynamic diagonal solitons and breathers in a deep-water wave basin. Our results confirm and prove the existence of such unique and coherent beams of a quasi-1D and short-crested wave group in a nonlinear dispersive medium.

## Methodology

Our theoretical framework is based on the space-(2D + 1) NLSE for deep-water waves (7). For a wave envelope ψ(x,y,t) with carrier wavenumber k along the x direction and carrier frequency $\omega =\sqrt{gk}$, we have

$$i\left(\frac{\partial \psi }{\partial t}+c_{g}\frac{\partial \psi }{\partial x}\right)-\lambda \frac{\partial^{2}\psi }{\partial x^{2}}+2\lambda \frac{\partial^{2}\psi }{\partial y^{2}}-\gamma {\left|\psi \right|}^{2}\psi =0,$$ [1]

where $\lambda =\frac{\omega }{8k^{2}}$, $\gamma =\frac{\omega k^{2}}{2}$, and g denotes the gravitational acceleration. At the leading order, it is known that $\frac{\partial \psi }{\partial t}≃-c_{g}\frac{\partial \psi }{\partial x}$. This relation can be used to write the equation to express the wave packet propagation in space along the spatial x coordinate to give a time-(2D + 1) NLSE (9)

$$i\left(\frac{\partial \psi }{\partial x}+\frac{1}{c_{g}}\frac{\partial \psi }{\partial t}\right)-\frac{\lambda }{c_{g}^{3}}\frac{\partial^{2}\psi }{\partial t^{2}}+2\frac{\lambda }{c_{g}}\frac{\partial^{2}\psi }{\partial y^{2}}-\frac{\gamma }{c_{g}}{\left|\psi \right|}^{2}\psi =0.$$ [2]

As the measurements are made at fixed positions along the flume, Eq. **2** can be used for experimental investigations. Now, we introduce the following transformation,

$$T=t\cos 𝜗-\frac{y}{c_{g}}\sin 𝜗,$$ [3]

with variable parameter 𝜗 that sets a special relation between time, t, and the spatial coordinate y. Then, the evolution equation for the new wave function, ψ(x,T), reads

$$i\left(\frac{\partial \psi }{\partial x}+\frac{1}{C_{g}}\frac{\partial \psi }{\partial T}\right)\;-\Lambda \frac{\partial^{2}\psi }{\partial T^{2}}-\Gamma {\left|\psi \right|}^{2}\psi =0,$$ [4]

with $C_{g}=c_{g}/\cos 𝜗$, $\Lambda =\lambda \left(1-3\sin^{2}𝜗\right)/c_{g}^{3}$, and $\Gamma =\gamma /c_{g}$. When the angle $|𝜗|<\sqrt{\arcsin\left(1/3\right)}≃35.26^{○}$ (15, 16), Eq. **4** is the standard (1D + 1) focusing NLSE that is known to be integrable (8, 26, 27). When 𝜗 ≠ 0, the envelope and the phase travel at a finite angle to each other.

From an experimental point of view, the boundary condition for the surface elevation η(x,y,t) at the wave maker, placed at x=0, can be described, to the leading order, by the expression

$$\eta \left(x=0,y,t\right)=\frac{1}{2}\left[\psi \left(0,T\right)\exp\left(-i\omega t\right)\;+\;c.c.\right],$$ [5]

where ψ(0,T) is the desired solution of the (1D + 1) NLSE in Eq. **4** and T is given by Eq. **3**. Eq. **5** is used for driving the wave maker.

**Fig. 1.**
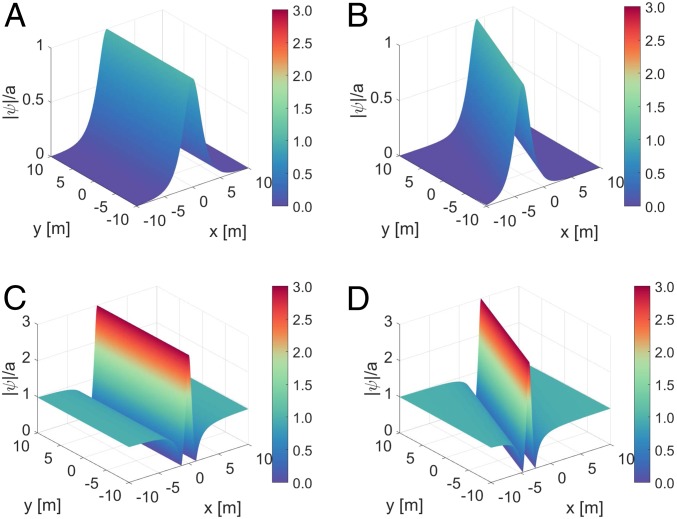
Normalized representation of a unidirectional as well as slanted NLSE envelope soliton and Peregrine breather for a carrier amplitude a=0.02 m and steepness ak=0.1 at t=0. (*A*) Unidirectional envelope soliton dynamics for $𝜗=0^{○}$. (*B*) Envelope soliton dynamics slanted by an angle of $𝜗=20^{○}$. (*C*) Unidirectional Peregrine breather dynamics for $𝜗=0^{○}$. (*D*) Peregrine breather dynamics slanted by an angle of $𝜗=20^{○}$.

To illustrate this type of universal and directional wave packet, in Fig. 1 we show an example of the dimensional shape of an envelope soliton and a Peregrine breather, as parameterized in refs. 26 and 28, with amplitude a=0.02 m propagating at zero diagonal angle (Fig. 1 *A* and *C*) and at an angle of $𝜗=20^{○}$ (Fig. 1 *B* and *D*) with respect to the carrier wave whose steepness is ak=0.1.

## Experimental Setup

The experiments were performed in a directional wave basin, installed at the University of Tokyo. Its dimensions are $50\times 10\times 5\;m^{3}$.

**Fig. 2.**
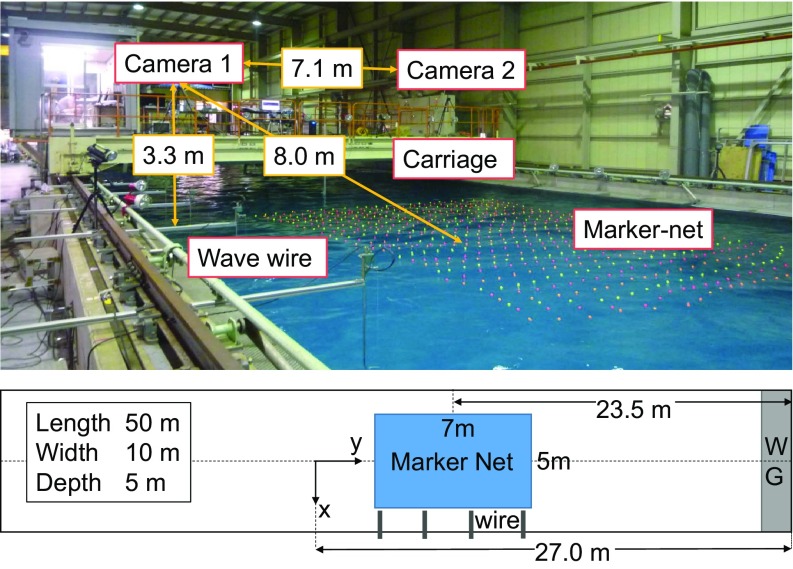
Experimental setup. The picture and the sketch show the dimensions of the flume, the location of the marker’s grid, and positions of the two stereo cameras.

To measure the directional wave evolution, a marker net was deployed at the center of the basin. The motion of the markers was recorded by two high-speed cameras with a resolution of 2,048×1,080 pixels at 100 frames per second. The two cameras are fully synchronized and are separated by 7.1 m distance across the tank in the y direction and positioned at 3.3 m from mean water level and about 10 m away from the center of the marker net in the x direction. Moreover, a series of wave wires were installed along the basin to follow the wave dynamics along the x coordinate. These were placed at 5.21 m, 9.20 m, 10.97 m, 14.01 m, 17.16 m, 20.15 m, 23.02 m, 27.04 m, 28.91 m, and 32.05 m from the directional plunger-type wave maker, which consists of 32 sections. Each plunger has a width of 32 cm. More details on the methodology adopted for the data acquisition can be found in ref. 29. A picture and a sketch of the experimental setup and the coordinate system adopted are depicted in Fig. 2.

We emphasize that due to the significant size of the digitally collected data, the stereo-reconstruction, that includes an interpolation process, is very challenging (29).

## Observations

**Fig. 3.**
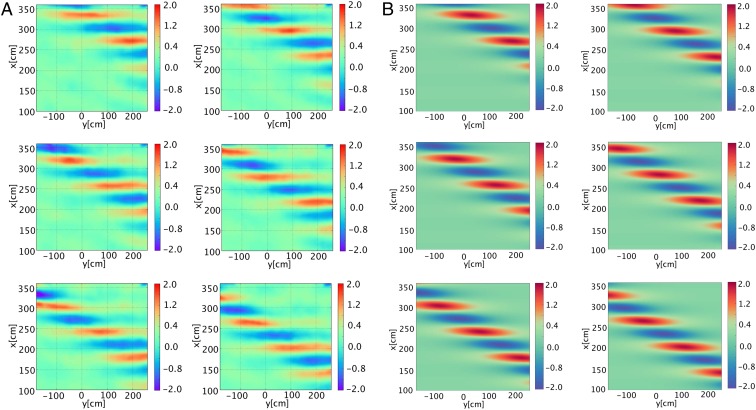
Evolution of a slanted envelope soliton propagating obliquely relative to the carrier wave. The amplitude, expressed in centimeters, is represented in color scale and the snapshots of the surface elevation are shown at intervals of time Δt=0.39 s. The parameters adopted are a=0.02 m, ak=0.2, and $𝜗=20^{○}$. (*A*) Stereo-reconstructed surface elevation of the deep-water soliton, propagating in the wave basin. (*B*) Analytical solution of the corresponding NLSE surface elevation of the slanted coherent structure following the NLSE **4** at the same time intervals.

The measured evolution of a sech-type envelope soliton (26) as well as the results obtained from the (2D + 1) NLSE prediction are illustrated in Fig. 3 *A* and *B*. Each of the corresponding six plots shows the oblique propagation of the localized and coherent structure with an angle of $𝜗=20^{○}$ with respect to the carrier wave with parameters ak=0.2 and a=0.02 for the time interval of Δt=0.39 s starting at $t_{0}=67.04$ s; that is, $t_{n}=t_{0}+n\Delta t$, n=0,…,5. Indeed, the direct comparison of the experimentally captured slanted envelope soliton in Fig. 3*A* with the analytical (2D + 1) NLSE prediction following Eq. **4** in Fig. 3*B* reveals a very good agreement. This becomes particularly clear when comparing the accuracy of the phase as well as group velocities of the soliton propagation in the two cases, their amplitudes, and particularly the short crest lengths of the waves resulting from the infinite extent as well as localization of the wave packet in the transverse direction in each time frame.

**Fig. 4.**
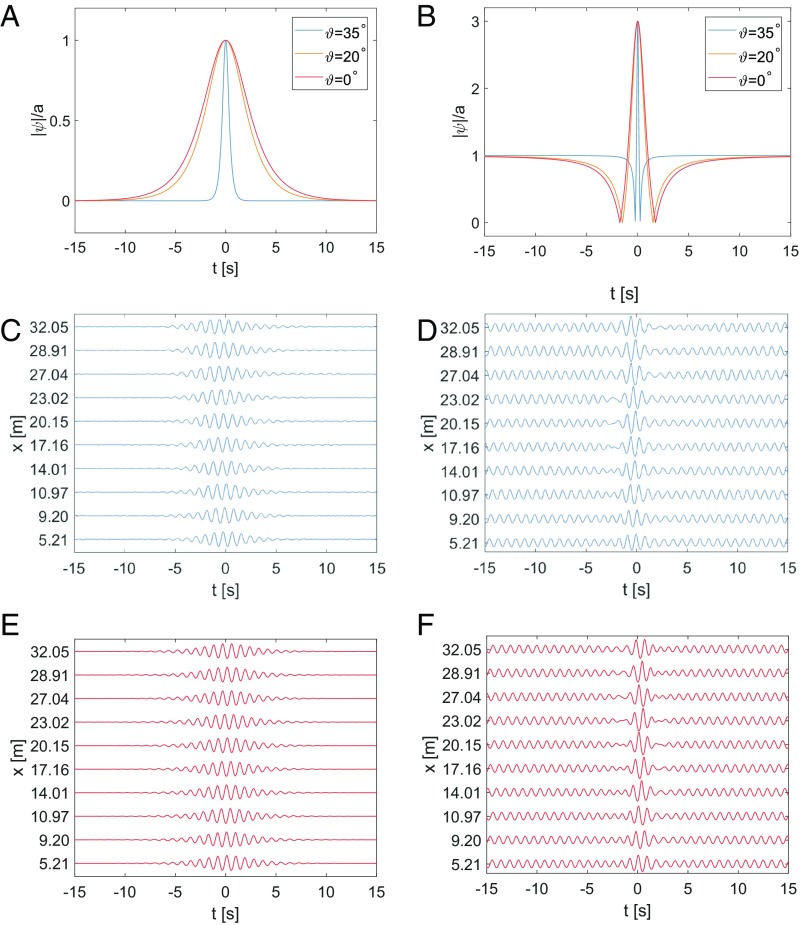
Influence of the obliqueness angle on the temporal width of two localized structures for representative carrier parameters of a=0.02 m and ak=0.1. (*A*) Theoretical envelope soliton surface for three different obliqueness angles. (*B*) Theoretical Peregrine breather surface for the same angle values as in A. (*C*) Temporal water surface profiles of the envelope soliton for $𝜗=20^{○}$ as measured in the basin. (*D*) Temporal water surface profiles of the Peregrine breather for $𝜗=20^{○}$ as measured in the basin. (*E*) Theoretical water surface profiles corresponding to *C*. (*F*) Theoretical water surface profiles corresponding to *D*.

The obliqueness angle 𝜗 also influences other parameters of the localized solutions. In particular, it affects the shape and the width of the soliton as well as the crest length of the directional wave field. Fig. 4*A* shows the case of the envelope soliton while Fig. 4*B* shows the Peregrine breather envelope profiles as functions in time for several angles of propagation $𝜗=0^{○}$, $𝜗=20^{○}$, and $𝜗=35^{○}$.

The soliton becomes thinner with increasing angle of propagation. The same applies to the Peregrine solution. Again, the profile of the solution compresses with increasing angle. In the case of periodic solutions, such as Akhmediev breathers or modulation instability in a general context, the period of the modulation will be also compressed.

In view of this angle-dependent compression, adjusted group velocity, and the complexities in the marker-net evaluation of the data, we restricted ourselves to the wave gauge measurements along the flume and the x direction.

Fig. 4*C* displays the evolution of the slanted envelope soliton for ak=0.1, a=0.02 m, and $𝜗=20^{○}$, while Fig. 4*E* shows the corresponding curves calculated theoretically.

The agreement between the experimental data and theoretical predictions is striking, especially when considering the preservation of the coherence and taking into account relatively large propagation distance of the soliton.

The oblique geometry also influences pulsating solutions localized in the propagation direction such as the Peregrine breather. Our equipment allowed us to generate them for a wide range of angles of propagation. Movies S1 and S2 show the evolution of the periodic Akhmediev and doubly localized breathers and corresponding legends for Movies S1 and S2 can be found in *SI Appendix*. Movies S1 and S2 clearly exemplify that the breather propagation direction differs from the carrier propagation direction just as in the case of the soliton. The difference of these directions is the major result of our observations. This discovery also demonstrates that localized, short-crested and directional water waves, particularly short-crested rogue waves, can be also described by a nonlinear framework.

Indeed, the Peregrine solution can be considered as the limit of the Akhmediev breather, the analytical and deterministic modulation instability model, when the period of the modulation tends to infinity (30, 31). Then, maxima of the periodic modulated structure are well separated and only one localized peak remains at the center. The temporal evolution of a slanted Peregrine solution measured in the experiment is shown and compared with the (2D + 1) NLSE predicted wave curves in Fig. 4 *D* and *F*, respectively. Again, comparison of the wave profiles in Fig. 4 *D* and *F* pair shows a remarkably good agreement between the measurements and the directional NLSE theory. The measured and calculated focusing distances, the maximal amplitudes, and the width of this localized and pulsating solution are all in excellent agreement at all stages of propagation.

## Discussion

Overall, our results reveal the existence of nonlinear solitary wave packets and breather beams, propagating obliquely to the direction of the wave field. This fact is confirmed by our experimental measurements for surface gravity water waves in a deep and directional water wave facility, installed at the University of Tokyo. Movies S1 and S2 clearly demonstrate and visualize this particular feature of nonlinear wave dynamics. The evolution of these packets is in excellent agreement with the (2D + 1) NLSE framework in regard to all wave features. A remarkable property of these particular localized wave packets studied here is their finite crest length. The latter can be observed by simply watching the ocean waves. The crest length and thus the transverse size of the waves is always limited. Now, it turns out that coherent waves with finite crest length might be a consequence of nonlinear beam dynamics. This is an important observation especially for the breather solutions, as this suggests that the nonlinearity is also a possible underlying mechanism for the actual finite-length–crested rogue wave events, complementing the linear superposition and interference arguments as has been generally suggested. Further studies using a fully nonlinear hydrodynamic approach (32, 33) may increase the accuracy of the description. These will characterize the ranges of accuracy of the approach; however, they will not add anything substantial to the concept. The serious implications of such wave packets in oceanography are an important aspect of our results (19). They include directional wave modeling, swell propagation, and diffraction as well as remote sensing of waves to name a few. Moreover, investigating wave breaking processes (34, 35) and prediction (36, 37) of extreme directional waves is also crucial for future application purposes. Since the effect can be explained by means of a general and universal theory for 2D nonlinear wave fields in dispersive environments, its further extensions can stimulate analogous theoretical, numerical, and experimental studies in 2D optical surfaces and multidimensional plasmas, among other relevant physical media, elevating our level of understanding of these phenomena.

## Supplementary Material

Supplementary File

Supplementary File

Supplementary File
