# Supplementary material for: Directional soliton and breather beams
Source: Proc Natl Acad Sci U S A. 2019 Apr 26;116(20):9759–63. doi: 10.1073/pnas.1821970116 (PMC6525507; doi:10.1073/pnas.1821970116)
Supplement: Supplementary File [file pnas.1821970116.sapp.pdf]

PNAS
www.pnas.org

Supplementary Information for

Directional soliton and breather beams

Amin Chabchoub, Kento Mozumi, Norbert Hoffmann, Alexander V. Babanin, Alessandro Toffoli, James N. Steer, Ton S. van den Bremer, Nail Akhmediev, Miguel Onorato, and Takuji Waseda

Corresponding Author Name.
E-mail: amin.chabchoub@sydney.edu.au

This PDF file includes:

Captions for Movies S1 to S2

Other supplementary materials for this manuscript include the following:

Movies S1 to S2

www.pnas.org/cgi/doi/10.1073/pnas.1821970116

Movie S1. Visual representation of the propagation of an oblique periodic Akhmediev breather rogue waves evolving in the wave flume. The recording clearly shows that the breather direction differs from the carrier propagation orientation. The carrier parameters adopted are $a = 0.01$ m and $ak = 0.1$ and the breather angle is $\vartheta = 20^{\circ}$ while the modulation frequency has been chosen to satisfy the maximal modulation instability growth rate.

Movie S2. Visual representation of the propagation of an oblique doubly-localized Peregrine breather extreme wave for the carrier parameters $a = 0.02$ m and $ak = 0.1$ with an oblique angle of $\vartheta = 20^{\circ}$. This movie also exemplifies the diagonal breather beam dynamics as well as the short-crested modulated and large-amplitude waves, located in the unstable wave packet.
